# Assessing SPECT/CT for the identification of cartilage lesions in the knee joint: A systematic review

**DOI:** 10.1016/j.ocarto.2025.100577

**Published:** 2025-01-30

**Authors:** Larissa Rix, Samuel Tushingham, Karina Wright, Martyn Snow

**Affiliations:** a The Robert Jones and Agnes Hunt Orthopaedic Hospital Foundation Trust, Oswestry, SY10 7AG, United Kingdom; b Centre for Regenerative Medicine Research, Keele University, Staffordshire, ST5 5BG, United Kingdom; c The Royal Orthopaedic Hospital, Birmingham, United Kingdom; d The Shrewsbury and Telford NHS Trust, Shrewsbury, United Kingdom

**Keywords:** Imaging, Diagnostics, Cartilage lesions, Knee pain, SPECT/CT, Nuclear medicine

## Abstract

**Background:**

Single-photon emission computerised tomography with conventional computer tomography (SPECT/CT) is an emerging technology which may hold clinical value for the identification of cartilage lesions in the knee joint. The intensity and distribution of SPECT/CT uptake tracer may identify physiological and structural information in the absence of structural change on other imaging modalities.

**Objectives:**

To systematically assess the utility of SPECT/CT in the detection of chondral lesions within the knee joint, in patients presenting with knee pain, with or without structural change.

**Results:**

PubMed, Science Direct, Web of Knowledge, and NHS databases were searched for English language articles focusing on the diagnostic value of SPECT/CT for knee chondral lesions and knee pain. Animal studies, cadaver studies, comparator radiological technique other than SPECT/CT or patients with a pathology other than knee chondral lesions were excluded. From the search, 11,982 manuscripts were identified, and screened for relevance. Seven studies were identified and scored low on QUADAS-2 bias review. SPECT/CT correlated with lesions found on other imaging modalities and during intraoperative assessment. Furthermore, in some cases, SPECT/CT out-performed other modalities in the detection of cartilage lesions.

**Conclusion:**

Evidence suggests SPECT/CT may be a useful tool for the detection and localisation of cartilage lesions, particularly in discrepant cases when there is an absence of lesions on other imaging modalities, or a lack of correlation with patients’ symptoms. Further studies are required to confirm the conclusions of this review.

## 1 Introduction

Anterior knee pain affects individuals of all age groups; however, it is one of the more common causes of orthopaedic pain seen in individuals <45 years of age [1]. Pain which intensifies in, or around, the anterior aspect of the knee, during weight bearing activities, is a prominent feature of the disorder [2]. Whilst multifactorial it is typically stated to occur through overuse, malalignment, trauma, and muscular imbalance, resulting over time in potential chondral damage [3]. When left untreated, these chondral lesions can develop into osteoarthritis (OA) [3]. Particularly in the young, the diagnosis can be difficult due to the absence of structural change on imaging confirming the origins of the pain.

The most widely used musculoskeletal imaging modalities are x-ray, CT, and MRI [4]. When diagnosing capsule, ligament, cartilage, and bone injuries related to patella dislocation, MRI has been demonstrated to be highly sensitive, compared to x-ray and CT, which focus mainly on bone [5]. Despite remaining the gold standard for the identification of chondral lesions, MRI is not a highly sensitive tool, with around 20 ​% of chondral lesions being mis-diagnosed, particularly in superficial lesions with <50 ​% chondral substance loss [6].

Single photon emission computed tomography (SPECT)/CT is a combination of the functional images produced from SPECT and anatomical images from CT [[7], [8], [9], [10]]. Functional images are generated from the administration of radioactive tracers which can then identify any metabolic change occurring [[7], [8], [9], [10], [11]]. SPECT/CT is sensitive to the changes that occur in osteoblastic metabolism. These metabolic changes can indicate stress within the subchondral bone, which has been implicated in pain generation [[7], [8], [9]]. The imaging modality is generally used for bone imaging and its major advantage over other nuclear medicine studies, is that it can evaluate precise anatomical and metabolic activity [12]. Specifically, the intensity and distribution of the metabolic tracer uptake on SPECT/CT has been shown to correlate with structural alignment features such as patellar tilt and patellar height. Thus, providing physiological and structural information [8,10].

SPECT/CT may provide valuable diagnostic information and identify radiological features absent on other imaging modalities, such as chondral lesions or areas of inferior cartilage quality and function in the knee joint, prior to any structural change. It thus has the potential to aid with the diagnosis and management of chondral lesions, particularly when MRI demonstrates no cartilage abnormalities [12]. Despite SPECT/CT showing high diagnostic accuracy for the assessment of late-stage knee OA or knee pain after arthroplasty, to our knowledge, no systematic review has been conducted to assess the utility of SPECT/CT in the detection of chondral lesions within the knee joint prior to structural change [13,14]. This review aims to highlight the potential of SPECT/CT as a diagnostic tool for chondral lesions within the knee joint in patients with knee pain, with or without a structural change.

## 2 Methods

### 2.1 Search strategy

An electronic database search was conducted using PubMed, Science Direct, Web of Knowledge, and NICE NHS database. The final search of the databases concluded December 2023 and included search terms relating to SPECT/CT and the knee joint. Abstracts and grey literature were not included unless full corresponding peer-reviewed study could be sought. The same keyword search approach was conducted for all database searches including:

(SPECT/CT).

(SPECT/CT) AND “chondral lesions”.

(SPECT/CT) AND “anterior knee pain”.

(SPECT/CT) AND “patellofemoral”.

(SPECT/CT) AND “knee”.

### 2.2 Study selection

Following an initial search of the databases, two independent reviewers selected relevant studies based first on title, then on abstract (L.R, S.T), through Rayyan software. Consensus was used to resolve disagreement between the two authors. In cases where consensus was not reached, a third reviewer was consulted (M.S). Relevant studies which were deemed appropriate, based on eligibility criteria, were further independently reviewed for inclusion using full text articles.

### 2.3 Eligibility criteria

Any article focusing on the diagnostic value of SPECT/CT for knee chondral lesions and knee pain were included. English language only articles were included to avoid loss of information in translation as English language was the first language of all the authors of the review and language resources, such as professional translators, were not available. Furthermore, any animal studies, cadaver studies, radiological technique other than SPECT/CT or patients with a pathology other than knee chondral lesions were excluded. Studies which included patients who had undergone arthroplasty were excluded.

### 2.4 Risk of bias and applicability

Once eligible studies were acquired, each study was independently assessed for bias by two reviewers (L.R, S.T) using the Quality Assessment of Diagnostic Accuracy Studies-2 (QUADAS-2) tool. When agreement on quality could not be reached, disagreement was resolved through consensus mediated by a third reviewer (M.S).

QUADAS-2 is comprised of four key domains: Patient selection; Index test; Reference standard; Flow and timing. Each of the domains was assessed in terms of the risk of bias, and the first three domains were also assessed in terms of concerns regarding applicability. To determine the judgement on the risk of bias, signalling questions were included in each domain. The signalling questions were answered as ‘yes’, ‘no’, or ‘unclear’, with ‘yes’ indicating low risk of bias and ‘no’ indicating high risk of bias. The risk of bias was then judged overall as ‘low’, ‘high’ or ‘unclear’.

### 2.5 Data collection

Data was collected by two independent reviewers (L.R, S.T) and collated into a table to ensure thorough evaluation of included articles. Data collected included participant characteristics inclusive of mean age, gender, primary knee complication, comparator technique, sample size, and article findings.

### 2.6 Data synthesis

Findings of the articles were synthesised narratively using the PRISMA extension for systematic reviews without meta-analysis. No standardisation or transformation method was used due to their being no common outcome measure between the included studies. Heterogeneity of the reported effects was not applicable.

Synthesis of the results were narrative and conclusions were drawn based on the repetitiveness of themes across the studies. Results table and synthesis were organised by gold standard comparator followed by author alphabetically.

## 3 Results

### 3.1 Study selection

**Fig. 1:**
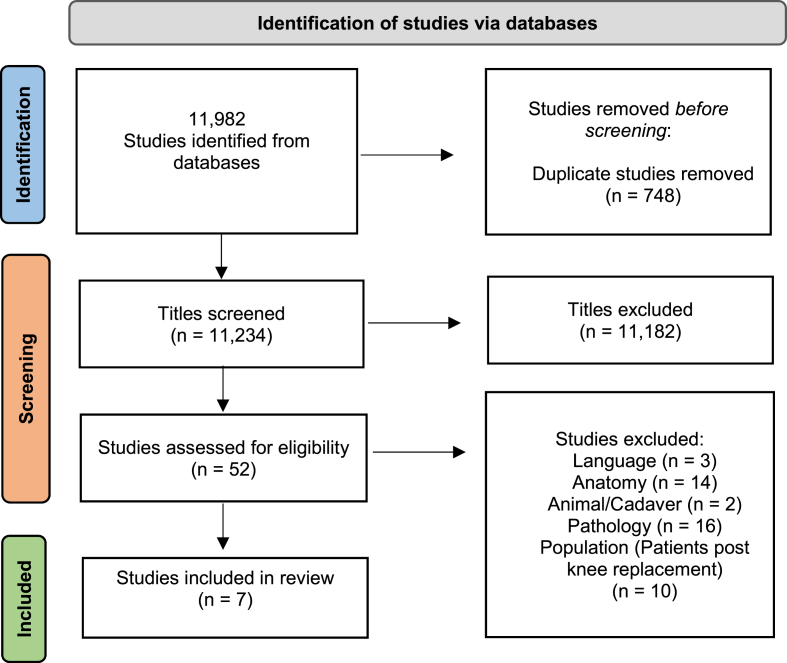
Flowchart of articles. The processing of 11,982 articles based on eligibility criteria to return 7 articles relevant for inclusion into review.

The systematic search revealed 11,982 studies to be screened. After screening the titles for relevance, removing duplicated papers, and applying the eligibility criteria, 52 studies were selected for further reviewing. Following full text review, 45 studies were excluded based on eligibility and 7 studies were considered eligible for this systematic review, as shown in the flowchart (Fig. 1).

### 3.2 Risk of bias and applicability

The risk of bias and applicability of the 7 included studies was assessed using QUADAS-2, as seen in Table 1. Of the 7 studies included, all studies scored a low overall risk of bias and applicability and were hence classed as high quality.

**Table 1:** Quality and applicability assessment of relevant articles.

Study Author	Study Design	Risk of Bias				Applicability			Support for Judgement
		Patient Selection	Index Test	Reference Standard	Flow and Timing	Patient Selection	Index Test	Reference Standard	
Ammann et al. [17]	PCSS	L	L	L	L	L	L	L	Low risk across all domains
An et al. [18]	RCS	U	L	H	L	L	L	L	High bias due to nuclear medicine technique (BS) used as reference test
Dordevic et al. [20]	RCS	L	L	L	L	L	L	L	Low risk across all domains
Koh et al. [16]	RCS	L	L	L	L	L	L	L	Low risk across all domains
Lu et al. [19]	RCS	U	L	L	L	L	L	L	Low risk across all domains
Maas et al. [21]	RCSS	L	L	L	L	L	L	L	Low risk across all domains
Ro et al. [15]	RCS	L	U	U	L	L	L	L	Unclear if index and reference test results were interpreted without prior knowledge

PCSS ​= ​Prospective cross-sectional study, RCS ​= ​Retrospective cohort study, RCSS ​= ​Retrospective cross-sectional study. L ​= ​Low grade, U ​= ​Unclear grade, BS ​= ​Bone Scintigraphy. All articles have 2 or less grades of unclear bias.

### 3.3 Study characteristics

Four hundred and thirty-three participants were included into the analysis across all 7 studies. The gold standard comparator varied amongst studies, in 22 ​% (95/433) clinical assessment was the comparator group, arthroscopic assessment via international cartilage regeneration and joint preservation society (ICRS) score was the comparator in 25 ​% (107/433), bone scintigraphy (BS) was the comparator in 33 ​% (143/433) and MRI was the comparator group in 20 ​% (88/433) (Table 2). Baseline characteristics of each study were extracted, and imputed into Table 3, inclusive of sample size, age, sex, gold standard comparator, and main inclusion criteria. Out of the studies included, only Ro et al., restricted the age range of participants to 40–65 years. The mean age ranged between 43 and 61 years of age for included articles [15]. Only 2 articles recruited more male than females, with the remaining 5 articles being female dominated. For all studies, knee pain was a primary inclusion criterion.

**Table 2:** Study comparator technique methodology and chondral lesion evaluation.

Study	Comparator Method	Comparator Definition	SPECT/CT Method	SPECT/CT Definition
Koh et al., 2015 [16]	Clinical Assessment NRS-11 Score	0: No pain 1-3: Mild pain 4-6: Moderate pain 7-10: Severe pain	1100 MBq 99m-Tc-HDP injection. SPECT/CT performed on Symbia T16.	FTU: Focal lesion distinguishable from back-ground activity. ITU: Inhomogeneous or diffuse uptake. NTU: Uptake that was not distinguishable from back-ground activity.
Ammann et al., 2019 [17]	Arthroscopic ICRS Cartilage Lesion Grade	Grade 0: Normal. Grade I: Soft indentation/superficial fissures. Grade II: Lesion <50 ​% of cartilage depth. Grade III: Lesion >50 ​% cartilage depth. Grade IV: Severely abnormal	700 MBq 99m-Tc-HDP injection. SPECT/CT performed on Symbia T16.	Maximum BTU values calculated on 11 anatomical regions using IntroSPECT customised software. For each region, the relative maximum BTU was calculated in relation to the BTU of the femur, which was classed as normal background BTU.
Ro et al., 2015 [15]	Arthroscopic ICRS Cartilage Lesion Grade	Grade 0: Normal. Grade I: Soft indentation/superficial fissures. Grade II: Lesion <50 ​% of cartilage depth. Grade III: Lesion >50 ​% cartilage depth. Grade IV: Severely abnormal	1100 MBq 99m-Tc-HDP injection. SPECT/CT performed on Infinia™ Hawkeye® 4.	Uptake grade 0: No uptake (equivalent to normal cancellous bone). Uptake grade 1: Higher than cancellous bone but lower than articular surface. Uptake grade 2: Uptake same as articular surface. Uptake grade 3: Uptake higher than articular surface.
An et al., 2021 [18]	740 MBq 99m-Tc-HDP injection. Bone Scintigraphy performed using dual headed gamma camera on NMCT/670 low-energy, high-resolution collimator.	Grade 0: No uptake. Grade 1: Mild uptake. Grade 2: Intense uptake.	740 MBq 99m-Tc-HDP injection. SPECT/CT performed on NMCT/670 scanner.	Grade 0: No uptake. Grade 1: Mild uptake. Grade 2: Intense uptake.
Lu et al., 2018 [19]	600 MBq 99-Tc-HDP injection. Bone Scintigraphy performed using dual headed gamma camera with low-energy, high- resolution collimator.	Detected: If a lesion could be visualised on the imaging modality. Localised: If the location of the lesion was determined. Characterised: If a single diagnosis could be made.	600 MBq 99-Tc-HDP injection. SPECT/CT performed using Precedence 16.	Detected: If a lesion could be visualised on the imaging modality. Localised: If the location of the lesion was determined. Characterised: If a single diagnosis could be made.
Dordevic et al., 2016 [20]	MRI with modified Noyes Grading Scale	Grade 0: Normal. Grade 1: Increased signal intensity of morphologically normal cartilage. Grade 2: A) Cartilage defect <50 ​% of articular surface thickness, or B) cartilage defect >50 ​% of articular surface thickness. Grade 3: Full-thickness defect.	500 MBq 99-Tc-HDP injection. SPECT/CT performed using Symbia T16.	Maximum uptake values for each anatomical region were recorded and calculated with reference to a reference region in the femoral shaft.
Maas et al., 2015 [21]	MRI with WORMS Score	Grade 0: Normal cartilage thickness and signal intensity. Grade 1: Normal cartilage Thickness/swelling with abnormal signal in ﬂuid-sensitive sequences. Grade 2: Partial cartilage defect <1 ​cm. Grade 2.5: Full-thickness defect <1 ​cm. Grade 3: Multiple grade 2 defects mixed with normal thickness/a grade 2 defect wider than 1 ​cm but <75 ​% of the region. Grade 4: Diffuse (>75 ​% of region) partial cartilage loss. Grade 5: Multiple cartilage loss (grade 2.5)/grade 2.5 lesion wider than 1 ​cm but <75 ​% of region. Grade 6: >75 ​% of region cartilage loss	740 MBq 99-Tc-HDP injection. SPECT/CT performed using Symbia T6 or T16.	Grade 0: Normal uptake with signal equal to adjacent bone of femur or tibia. Grade 1: Moderately elevated uptake with focal signal inhomogeneity. Grade 2: Severely elevated uptake with intense focal uptake with background suppression in non-affected bone.

NRS-11 ​= ​Numeric Rating Scale-11, FTU ​= ​Focal Tracer Uptake, ITU ​= ​Irregular Tracer Uptake, NTU ​= ​No Tracer Uptake, ICRS ​= ​International cartilage regeneration and joint preservation society, BTU ​= ​Bone Tracer Uptake, MRI ​= ​Magnetic resonance imaging, WORMS ​= ​whole organ magnetic resonance imaging score.

**Table 3:** Study and patient characteristics of included articles.

Study Author	Gold Standard	Sample Size	Mean Age (y)	Sex		Primary Inclusion Criteria	Findings
				M	F		
Koh et al., 2015 [16]	Clinical assessment	95	59	33	62	Knee pain	Those with tracer uptake of lesions coincided with the area of pain complaint. Lesions were found by SPECT/CT in 4 patients outside the area of complaint.
Ammann et al., 2019 [17]	Arthroscopic assessment	33	61	11	22	Chronic knee pain >6 months	Tracer uptake correlated with ICRS score.
Ro et al., 2015 [15]	Arthroscopic assessment	74	54	23	51	Knee pain with no known cause >8 weeks	Tracer uptake correlated with ICRS score. Higher uptake in PFJ of patients with chronic AKP is predictive of poor response to conservative management.
An et al., 2021 [18]	Bone Scintigraphy	104	58.3	19	85	Knee pain	15 ​% of lesions scored higher uptake on SPECT/CT and the total number of lesions identified was higher for SPECT/CT.
Lu et al., 2018 [19]	Bone Scintigraphy	39	43	14	25	Knee pain and x-ray, BS, and SPECT/CT	SPECT/CT detected, localised, and characterised 100 ​% of lesions, cf. BS, which detected 91 ​%, localised 42 ​% and characterised 52 ​% respectively.
Dordevic et al., 2016 [20]	MRI	63	49.2	42	21	At least 1 chondral lesion	SPECT/CT significantly correlated with grade and size of lesions as MRI.
Maas et al., 2015 [21]	MRI	25	47.7	14	11	Chronic knee pain >6 months	Significant uptake by SPECT/CT with increasing cartilage WORMS score. SPECT/CT found increased uptake in 29 regions where MRI showed normal cartilage.

M ​= ​Male, F ​= ​Female; MRI ​= ​Magnetic Resonance Imaging; N ​= ​Number of participants; ICRS ​= ​International cartilage regeneration and joint preservation society; PFJ ​= ​Patellofemoral joint; AKP ​= ​Anterior knee pain; WORMS ​= ​Whole organ MRI scoring; BS ​= ​Bone scintigraphy.

### 3.4 Clinical evaluation

Only Koh et al., compared SPECT/CT to clinical assessment in 95 consecutive patients for knee pain. Knee pain was defined according to duration and was scored using a numeric rating scale (NRS-11) [16].

Of the 95 patients included, 32 patients had focal tracer uptake (FTU) on SPECT/CT, 39 patients had irregular tracer uptake (ITU) and 24 patients had no tracer uptake. Location of the chondral lesion in patients with FTU on SPECT/CT coincided with the initial area of complaint. However, in 4 patients, SPECT/CT detected additional chondral lesions with FTU, outside of the main area of complaint, of which MRI confirmed as grade 4 lesions [16]. In patients with chronic knee pain (n ​= ​57), 42 patients (74 ​%) exhibited tracer uptake on SPECT/CT and 15 patients (26 ​%) had no tracer uptake. In those chronic knee pain patients with FTU, there was no significant correlation between tracer uptake and initial NRS-11 score (r ​= ​0.014). In contrast, this pain score increased when ITU increased (r ​= ​0.436, *p* ​= ​0.008) [16].

### 3.5 Intraoperative assessment

Ammann et al., found that tracer uptake on SPECT/CT significantly correlated with ICRS score for the trochlea (r ​= ​0.35, *p* ​< ​0.05) and the medial patella facet (r ​= ​0.44, *p* ​< ​0.05) [17]. There was also significant moderate correlation for SPECT/CT uptake and ICRS score for the medial femoral condyle (r ​= ​0.59, *p* ​< ​0.001) and anterior medial tibial plateau (r ​= ​0.57, *p* ​< ​0.001). No significant correlation was seen in the lateral compartment. Additionally, it was found that the medial compartment had the highest uptake on SPECT/CT (4.4 ​± ​3.3) and ICRS mean score (2.3 ​± ​1.18), which authors stated was of no surprise due to the varus alignment of the patients’ legs [17]. The patellofemoral compartment showed the second highest uptake (3.0 ​± ​2.0), and the lateral compartment had the lowest uptake (2.6 ​± ​1.3) [17]. In comparison, the lateral compartment had the second highest ICRS mean score (1.92 ​± ​0.84), followed by the patellofemoral compartment that had the lowest ICRS mean score (1.81 ​± ​0.75) [17]. The discrepancy between the imaging modalities was due to only grade I and II lesions being present in these compartments, and the low accuracy of ICRS scoring system for such lesions.

Ro et al., compared ICRS score of lesions to tracer uptake on SPECT/CT in the patella and trochlea of anterior knee pain patients [15]. A grade 1 tracer uptake was seen in the patella and femoral trochlea for all 74 patients [15]. In addition, those patients who demonstrated a higher uptake in the patella were more likely to be non-responders to activity modification (*p* ​= ​0.009) [15]. The positive predictive value in non-response to treatment for a grade 2/3 patella uptake on SPECT/CT was 62–67 ​%, compared to only 24–25 ​% in grade 0/1 uptake. In non-responders to treatment, when the patella was subsequently assessed arthroscopically, the tracer uptake had good agreement with the ICRS score of the lesion (γ ​= ​0.625) [15]. In contrast, the agreement between the trochlea tracer uptake and the ICRS score of the lesion was only ‘fair’ (γ ​= ​0.397) [15]. The median uptake in the patella was found to be higher in the non-responder group (median ​= ​1.29) compared to the responder group (median ​= ​1) but there was no difference in the mean uptake of the trochlea for the two groups [15].

### 3.6 Planar imaging

An et al., studied 104 patients to evaluate knee pain using tracer uptake on SPECT/CT compared to BS [18]. Around 80 ​% of visual uptake grades were similar between BS and SPECT/CT, but 15.5 ​% of grades scored higher using SPECT/CT [18]. The total number of regions per patient with grade 1 or higher uptake grades, was significantly higher for SPECT/CT (3.3 ​± ​2.0) than BS (2.4 ​± ​2.3) (*p* ​< ​0.001). In the patellofemoral joint, 23 lesions (11 ​%) with grade 1 or higher uptake were found using SPECT/CT compared to only 14 lesions (6.9 ​%) on BS [18]. When comparing the mean standardised uptake value on SPECT/CT with the mean quantified uptake value on BS, there was a significant moderate correlation in all knee regions (r ​= ​0.58, *p* ​< ​0.0001).

Lu et al., analysed 39 patients who presented in clinic with knee pain and had undergone BS and SPECT/CT imaging [19]. To reduce the chance of false positives, only symptomatic lesions were evaluated. Of 105 lesions, BS detected 91 ​% of lesions compared to SPECT/CT which detected 100 ​% of lesions [19]. Moreover, for those lesions detected by both modalities (n ​= ​96), 44 lesions were due to OA, and SPECT/CT accurately characterised 100 ​% of the lesions compared to 97.9 ​% by BS. In addition, a subset analysis of 9 knees which had coexisting conditions such as patellar maltracking (n ​= ​6), osteochondral lesions (n ​= ​2) and loose bodies (n ​= ​1), these conditions were all detected by SPECT/CT alongside the chondral lesions, in comparison to BS which could only detect the lesions [19]. In patients with knee pain (n ​= ​39), SPECT/CT localised 100 ​% of lesions compared to 38.5 ​% using BS (*p* ​< ​0.001). For this same cohort, SPECT/CT was able to obtain a diagnosis in 92.3 ​% of lesions compared to BS which could only diagnose 23 ​% (*p* ​< ​0.001) [19].

### 3.7 Magnetic resonance imaging

Dordevic et al., assessed the correlation between tracer uptake of lesions on SPECT/CT and the size and severity of chondral lesions in the knee detected by MRI using Noyes grading scale [20]. Those with a grade 0 lesion on MRI correlated with a mean tracer uptake of 1.64 ​± ​0.95, grades 1 and 2 correlated with mean tracer uptake of 2.95 ​± ​2.07 and grades 3 and with a mean tracer uptake of 3.61 ​± ​2.18. Significant differences in tracer uptake were found between all chondral lesion grades (*p* ​< ​0.001–0.002) [20]. Zones where no chondral lesions were present on MRI showed significantly lower tracer uptake on SPECT/CT compared to zones with lesions present of all sizes and grades (<1 ​cm^2^, *p* ​= ​0.45; 1–4 ​cm^2^, *p* ​< ​0.001; >4 ​cm^2^, *p* ​< ​0.001) [20]. In those patients presenting with higher grades of chondral lesions (grade 3 and 4) and larger chondral lesion size (>4 ​cm^2^) on MRI significantly higher tracer uptake was observed on SPECT/CT compared to smaller sizes (<1 ​cm^2^, *p* ​= ​0.011; 1–4 ​cm^2^, *p* ​= ​0.004) [20]. No significant differences were shown in tracer uptake when comparing lesions <1 ​cm^2^ or for patients with lower grades (1 and 2) [20].

Maas et al., also investigated the association between chondral lesions assessed with MRI using whole-organ MRI score (WORMS) and SPECT/CT. Lesions were found to be relatively equally distributed amongst the regions for both imaging modalities [21]. When comparing the uptake level on SPECT/CT to MRI, for all 162 regions, a significant uptake on SPECT/CT with increasing cartilage score was found (*p* ​< ​0.0001) [21]. The lowest mean uptake was in regions in which no cartilage lesions were present (WORMS grade 0), and the highest mean uptake was seen in regions with severe cartilage lesions (WORMS 5) [21]. The patella (66 ​%) and medial femoral condyle (56 ​%) showed the highest prevalence of cartilage lesions on MRI whilst medial tibia showed lowest (30 ​%) [21]. In comparison, the highest prevalence of uptake on SPECT/CT was seen in the patella (63 ​%) and lateral tibia (59 ​%), whilst the lowest was seen in the medial and lateral femoral condyles (41 ​%) [21]. The two highest uptakes on SPECT/CT were associated with grades that describe full thickness lesions. Interestingly, increased uptake on SPECT/CT was found in 29 lesions (35 ​%) with normal cartilage (WORMS 0), and in 3 out of 4 (75 ​%) of grade 1 cartilage lesions [21]. On comparing the uptake scores per joint (uptake sum), a moderate positive significant correlation was found between uptake sum and WORMS (r ​= ​0.42, CI 0.01–0.67, *p* ​= ​0.028).

## 4 Discussion

SPECT/CT may be valuable for the detection of chondral lesions in the knee joint, particularly for those who cannot undergo MRI, or do not show any structural changes on MRI but present with chronic knee pain refractory to non-surgical therapies. SPECT/CT appears to be a highly sensitive, specific diagnostic tool compared to other imaging modalities [9,22]. Whilst SPECT/CT carries additional radioactive implications, it is important to view SPECT/CT as a complementary modality, rather than competitive.

MRI is recognised as the gold standard technique for chondral lesion detection; however, the question remains as to whether SPECT/CT would have additional diagnostic value. One major limitation of MRI is that the measurements acquired are subjective to the observer and therefore can introduce variability. MRI is also believed to underestimate the severity of the cartilage lesions and the diagnostic accuracy of grade I-III chondral lesions have been shown to be limited [23]. A recent meta-analysis revealed that MRI had a 75 ​% sensitivity and 95 ​% specificity for the diagnosis of chondral lesions in the knee, with the probability of having chondral lesions with a negative MRI being 22 ​% [24]. It is in this group where SPECT/CT can potentially play a role. SPECT/CT measures the intensity and the distribution of tracer uptake, which is a more objective, reproducible method and is not observer dependent. Maas et al., reported a difference between MRI and SPECT/CT for 35 ​% of regions with normal cartilage, whereby increased uptake was detected by SPECT/CT [21]. This is likely a consequence of patients being exposed to higher loads, given the known association between tracer uptake at sites of increased loading [8,25]. Hirschmann et al. demonstrated that the intensity and distribution of the tracer uptake on SPECT/CT correlated with mechanical overload of the knee joint, with resultant increased shear stress on the cartilage [8]. Hirschmann et al. also determined that SPECT/CT may be useful for the differentiation between chondral lesions in the knee joint or overloading as the cause of knee pain [8]. SPECT/CT has the ability to detect changes in the activation of osteoblastic metabolism. These metabolic changes are indicators of subchondral bone stress, which has been associated with pain [[7], [8], [9]]. Given tracer uptake is an objective measure of increased load, positive findings have the potential to be a more functional representation of osteochondral health and thus more clinically relevant. A major advantage of SPECT/CT is the additional element of CT imaging. Without CT, it is difficult to distinguish whether the uptake is from the soft tissue or the bone, due to the proximity of the structures [19]. CT also allows the imaging of potential anatomical abnormalities, such as tibial tubercle malalignment, which cause or contribute to chondral lesions through high loading. Thus, in addition to being a valuable diagnostic tool, it can also aid with decisions regarding the need for off-loading osteotomies [19].

MRI is reported to inconsistently correlate with the patients’ reported symptoms [17]. The findings of this review are reflective of those found by Hirschmann et al., who recommend the use of SPECT/CT in cases where plain radiography and MRI do not correlate with clinical observations [14]. In patients with chronic knee pain, tracer uptake was seen on SPECT/CT in 73 ​% of patients, and in 4 patients, SPECT/CT detected additional lesions outside of the initial area of complaint, confirmed to be grade 4 lesions by MRI [16]. SPECT/CT detected (visualised) 100 ​% and characterised (diagnosed) 92 ​% of lesions, for patients with chronic knee pain, compared to poorer accuracy of 38.5 ​% visualised using BS [16]. The potential of tracer uptake to detect early cartilage changes has been demonstrated in small animal models of OA. One animal study found that tracer uptake in the meniscectomised guinea pig demonstrated changes associated with pathologic cartilage, suggesting that focal uptake can be an indicator of the location of pain [26]. In addition, when irregular uptake increased, pain score similarly increased, suggesting that the intensity of ITU relates to the severity of the pain [16]. Thus, giving confidence to the idea that SPECT/CT may be a more useful tool for detecting chondral lesions before the onset of OA [6].

There has been limited research conducted to assess whether tracer uptake on SPECT/CT can predict the likely outcome after treatment [13]. Ro et al., addressed this and demonstrated that patients with higher tracer uptake on SPECT/CT within the patella were more likely to be non-responders to conservative management [15]. In contrast, Koh *et* al., found that patients with focal uptake in the knee on SPECT/CT had a better response to conservative treatment than those with no uptake [16]. Additionally, uptake on SPECT/CT, either FTU or ITU, showed no significant improvement in outcome post-surgical treatment [16]. Hirschmann et al. found a clinically relevant change in proposed treatment before and after conducting a SPECT/CT scan for 83 ​% (19/23) patients with arthroplasty revision [27]. Therefore, SPECT/CT may have the ability to predict treatment outcome for patients with cartilage pathology, however it may be that the more focused the tracer uptake is in the area, the better the response, rather than the amount of tracer uptake. More studies assessing treatment response would be beneficial.

SPECT/CT appears to be a highly sensitive and specific diagnostic tool compared to other imaging modalities for the detection of chondral pathology within the knee joint [14,22]. However, SPECT/CT does carry additional radioprotective implications, with an average dose of 7 ​mSv per SPECT/CT scan, giving around a 1 in 5000 additional risk of cancer per scan [28]. However, the average person in the UK is exposed to 1.5–7.5 ​mSv annually, regardless of SPECT/CT scan [28]. A combination of MRI and SPECT/CT has the potential to assist with the identification of patients with lesions, which are not visible on MRI, or for those who cannot undergo MRI, who would likely benefit from offloading treatment procedures.

## 5 Limitations

A consensus on the interpretation of tracer uptake value needs to be reached regarding which tracer uptake measurement is clinically relevant, i.e., does FTU demonstrate location of chondral lesion or pain intensity of chondral lesion, and what threshold value for the tracer uptake is relevant for each knee region, to provide optimal SPECT/CT methodologies.

The language was restricted to English due to all authors' first language being English and the additional time and cost of employing a translator. However, we acknowledge that relevant information may have been missed through restricting the search and that potential language bias may have been incorporated through this. Similarly, the exclusion of studies for which relevant data could not be obtained, such as from conference abstracts, may have led to publication bias.

When undertaking a scope of the literature prior to the review, it became evident that the review would have to be conducted in a narrative format as most studies did not sufficiently detail diagnostic results required for meta-analysis. Therefore, the review was not registered with PROSPERO in compliance with their guidelines. Whilst it is not a mandatory requirement to register reviews in PROSPERO, we view this as a limitation to the review. To ensure no duplication of review, the records of similar registered reviews in PROSPERO were checked prior to conducting the review. Whilst we have narratively deciphered the findings and conclusions of the included data, it is recognised that no definitive conclusion can be made regarding whether SPECT/CT should be implemented in the clinical decision-making process. Future studies that can be obtained to undertake a meta-analysis of the data would strengthen the confidence in the results.

Studies comparing uptake pre- and post-surgical intervention and clinical outcome are needed to determine SPECT/CT role in clinical decisions. Histological data to characterise the cartilage and bone health would give insight into tracer uptake pattern and meaning.

All the studies included in this review did not assess diagnostic accuracy such as sensitivity and specificity. Therefore, conclusions were based on the available statistical data from each article. More robust studies that determine the diagnostic accuracy of the modality for detecting chondral lesions prior to structural change on MRI would be beneficial.

## 6 Conclusion

The consensus of the literature supports that SPECT/CT is a beneficial imaging modality for the detection and localisation of chondral lesions of the knee, even prior to the presence of structural change on MRI. SPECT/CT may have a potential role in guiding treatment decisions or predicting treatment response. To determine this, studies which compare uptake pre- and post-surgical intervention and its relationship to clinical outcome are needed. The inclusion of histological data to characterise the cartilage and bone health would also give insight into tracer uptake pattern and meaning. Further investigation is required to undertake more robust studies that utilise SPECT/CT in daily practise to determine the diagnostic capacity of the imaging tool.

## Informed consent

None.

## Author contributions

L.R undertook all aspects of the review. S.T acted as second reviewer. M.S acted as consensus reviewer and provided clinical input. K.W provided biological expertise and helped with editing of manuscript.

## Ethical approval

None.

## Funding

The Orthopaedic Institute Ltd (RGP 179) and Versus Arthritis Tissue Engineering Centre (21,156). For the purposes of open access, the author has applied Creative Commons Attribution (CC-BY) licence to any accepted author manuscript version arising from this submission.

## Declaration of competing interest

The authors declare no conflicts of interest.
